# Pancreatic duct occlusion with polychloroprene-based glue for the management of postoperative pancreatic fistula after pancreatoduodenectomy—an outdated approach?

**DOI:** 10.3389/fsurg.2024.1386708

**Published:** 2024-04-05

**Authors:** Sheraz Yaqub, Bård Røsok, Ivar Prydz Gladhaug, Knut Jørgen Labori

**Affiliations:** ^1^ Department of Hepato-Pancreato-Biliary Surgery, Oslo University Hospital, Oslo, Norway; ^2^ Institute of Clinical Medicine, University of Oslo, Oslo, Norway

**Keywords:** duct occlusion, postoperative pancreatic fistula, pancreatoduodenectomy, pancreatic surgery, reoperation

## Abstract

**Background:**

Managing postoperative pancreatic fistula (POPF) presents a formidable challenge after pancreatoduodenectomy. Some centers consider pancreatic duct occlusion (PDO) in reoperations following pancreatoduodenectomy as a pancreas-preserving procedure, aiming to control a severe POPF. The aim of the current study was to evaluate the short- and long-term outcomes of employing PDO for the management of the pancreatic stump during relaparotomy for POPF subsequent to pancreatoduodenectomy.

**Methods:**

Retrospective review of consecutive patients at Oslo University Hospital undergoing pancreatoduodenectomy and PDO during relaparotomy. Pancreatic stump management during relaparotomy consisted of occlusion of the main pancreatic duct with polychloroprene Faxan-Latex, after resecting the dehiscent jejunal loop previously constituting the pancreaticojejunostomy.

**Results:**

Between July 2005 and September 2015, 826 pancreatoduodenectomies were performed. Overall reoperation rate was 13.2% (*n* = 109). POPF grade B/C developed in 113 (13.7%) patients. PDO during relaparotomy was performed in 17 (2.1%) patients, whereas completion pancreatectomy was performed in 22 (2.7%) patients. Thirteen (76%) of the 17 patients had a persistent POPF after PDO, and the time from PDO until removal of the last abdominal drain was median 35 days. Of the PDO patients, 13 (76%) patients required further drainage procedures (*n* = 12) or an additional reoperation (*n* = 1). In-hospital mortality occurred in one patient (5.9%). Five (29%) patients developed new-onset diabetes mellitus, and 16 (94%) patients acquired exocrine pancreatic insufficiency.

**Conclusions:**

PDO is a safe and feasible approach for managing severe POPF during reoperation following pancreatoduodenectomy. A significant proportion of patients experience persistent POPF post-procedure, necessitating supplementary drainage interventions. The findings suggest that it is advisable to explore alternative pancreas-preserving methods before opting for PDO in the management of POPF subsequent to pancreatoduodenectomy.

## Introduction

Postoperative pancreatic fistula (POPF) is a well-known and challenging complication following pancreatoduodenectomy. POPF is characterized by a high rate of morbidity and mortality. Percutaneous drainage is the most common primary intervention for severe POPF (1). In selected cases, a reoperation is deemed necessary to mitigate POPF. During relaparotomy, several strategies are feasible: completion pancreatectomy, disconnection of anastomosis with preservation of the pancreatic remnant, establishing internal or external wirsungostomy, salvage pancreaticogastrostomy, or optimal surgical drains along the pancreas (2–4). Current evidence suggests that opting for a pancreas-preserving procedure is a more favorable choice than completion pancreatectomy in patients requiring a relaparotomy (5). Prophylactic pancreatic duct occlusion (PDO) has been explored as a substitute to pancreaticojejunostomy in pancreatoduodenectomy, aiming to reduce the risk of POPF (6–8). Several techniques of pancreatic stump occlusion have been described (6). Some centers also consider PDO in post-pancreatoduodenectomy reoperations as a pancreas-preserving procedure, aiming to control severe POPF (7, 9). The aim of the current study was to evaluate the short- and long-term outcomes of PDO in managing the pancreatic remnant during a relaparotomy for POPF following pancreatoduodenectomy.

## Methods

This was a retrospective review of consecutive patients undergoing pancreatoduodenectomy at Oslo University Hospital between July 2005 and September 2015. Patients who underwent PDO at reoperation to treat a POPF were identified from the institutional pancreatic database. Hospital records and pathology reports were reviewed. Postoperative complications were assessed according to the Clavien-Dindo classification. The Clavien-Dindo grading was used to calculate the Comprehensive Complication Index (CCI), by means of the online tool. POPF, including persistent POPF after PDO, was defined and graded according to criteria set by the International study Group on Pancreatic Fistula (10). The hospital review board approved the study (19/04710) according to the general guidelines provided by the Regional Ethics Committee. The manuscript was completed in accordance with the STROBE statement. Duration of hospital stay was calculated from the day of pancreatoduodenectomy until discharge. Ninety-day mortality and in-hospital mortality were defined as death within 90 days after surgery or during hospital stay, respectively. The surgical procedure consisted of pancreatoduodenectomy with standard lymphadenectomy. Pylorus-preserving pancreatoduodenectomy has been the standard approach since 2012, whereas a classic Whipple procedure was preferred between 2005 and 2011 (11). The pancreaticojejunostomy was performed in an end-to-side, two-layer, duct-to-mucosa technique (12). PDO was performed at the discretion of the treating surgeon. Pancreatic stump management during relaparotomy consisted of occlusion of the main pancreatic duct by polychloroprene Faxan-Latex (Oslo University Hospital Pharmacy, Oslo, Norway), after resecting the dehiscent jejunal loop previously anastomosed to the pancreas. In addition, the pancreatic duct was occluded at the resection margin with a non-absorbable U-suture. Faxan-Latex was no longer available in our institution from September 2015.

Descriptive statistics were collected and reported as a whole number (percentage) and median (range or interquartile range). Statistical analysis was carried out using IBM SPSS Statistics, Version 29.0.

## Results

Between July 2005 and September 2015, 826 pancreatoduodenectomies were performed, and 113 (13.7%) patients developed POPF grade B/C. Completion pancreatectomy was performed in 22 (2.7%) patients. PDO with polychloroprene Faxan-Latex during relaparotomy was performed in 17 (2.1%) patients. Baseline surgical and histopathological characteristics of these 17 patients are presented in Table 1. There were 8 male and 9 female with a median age of 71 years (range 63–79). Ten patients had periampullary adenocarcinoma (distal bile duct, ampullary, duodenal), two patients had pancreatic ductal adenocarcinoma, three patients had premalignant tumors, one patient had a neuroendocrine tumor, and one patient had chronic pancreatitis. Main outcomes are presented in Table 2. Hospital length of stay (LOS) was median 41 days. Thirteen (76%) patients had a persistent POPF after PDO, and time from PDO to removal of the last abdominal drain was median 35 days. These 13 patients required additional drainage procedures (*n* = 12) or a re-operation (*n* = 1). CCI was median 54, 90-day mortality was zero, whereas in-hospital mortality occurred in one patient (5.9%) after 135 days. No patients underwent a subsequent completion pancreatectomy. Postoperative new-onset exocrine or endocrine insufficiency occurred in 16 (94%) and 5 (29%) patients, respectively. In addition, two surgical complications were identified. One patient developed an intraabdominal abscess close to the pancreatic tail 17 months after the time of PDO, and one patient experienced perforation of the glue and a baby-feeding tube left behind in the pancreatic duct during PDO, to the jejunal loop, re-establishing a connection between the pancreatic duct and the jejunum.

**Table 1:** Baseline, surgical and histopathological characteristics in patients treated with pancreatic duct occlusion.

Characteristics	*N* = 17
Age, median (range), years	71 (63–79)
Sex, *n* (%)	
Male	8 (47)
Female	9 (53)
Comorbidity, *n* (%)^a^	
Cardiovascular	8 (47)
Diabetes mellitus	2 (12)
Hypertension	5 (29)
Chronic pulmonary disease	2 (12)
Other	8 (47)
ASA classification on admission, *n* (%)	
ASA I	2 (12)
ASA II	7 (41)
ASA III	8 (47)
BMI, median (range)	22.7 (17.8–29.5)
Preoperative biliary stent, *n* (%)	8 (47)
Details on pancreatoduodenectomy, *n* (%)	
Pylorus-preserving	8 (47)
Laparoscopic	1 (5)
Portal/Superior mesenteric vein reconstruction	2 (12)
Additional organ resection	2 (12)
Operative time, median (range), minutes	318 (250–504)
Estimated blood loss, median (range), ml	400 (50–3,000)
Red blood cell transfusion	4 (24)
Histopathology, *n* (%)	
Distal cholangiocarcinoma	6 (35)
Pancreatic ductal adenocarcinoma	2 (12)
Periampullary tubulovillous adenoma (high grade dysplasia)	2 (12)
Ampullary adenocarcinoma	2 (12)
Duodenal adenocarcinoma	1 (6)
Duodenal neuroendocrine carcinoma	1 (6)
Pancreatic neuroendocrine tumor	1 (6)
Intraductal papillary mucinous neoplasm (low grade dysplasia)	1 (6)
Chronic pancreatitis	1 (6)

Percentages might not sum to 100 as a result of rounding.

ASA, American society of anesthesiologists; BMI, body mass index.

^a^ Seven patients had multiple comorbidities.

**Table 2:** Main outcomes in patients treated with pancreatic duct occlusion.

Outcome	*N* = 17
Disease severity 24 h before time of duct occlusion	
Level of care, *n* (%)	
Intensive care unit	5 (29)
Ward	12 (71)
Organ failure, *n* (%)	
Single organ^a^	1 (6)
Multiorgan^b^	3 (18)
Time from primary operation to duct occlusion, median (range), days	9 (2–40)
Main cause of reoperation	
Abdominal pain plus CT findings^c^ or leukocytosis/elevated CRP	7
Post pancreatectomy hemorrhage	3
Fluid collection around PJ on CT, leukocytosis/elevated CRP, and clinical Deterioration	4
Fluid collection around PJ on CT, leukocytosis/elevated CRP, clinically Stable	1
Intestinal content on drain, clinically stable	2
Previous interventions, *n* (%)^d^	
Reoperation	2 (12)
Drainage procedure	5 (29)
None	10 (59)
Later interventions, *n* (%)	
Reoperation	1 (6)
Drainage procedure	12 (71)
None	4 (24)
Persistent pancreatic fistula after duct occlusion, *n* (%)	13 (76)
Time from duct occlusion until removal of last abdominal drain, median (range), days	35 (3–180)
Time from duct occlusion until removal of abdominal drain, days, *n* (%)	
1–15	3 (18)
15–30	4 (24)
30–45	6 (35)
46–90	1 (6)
91–180	3 (18)
Time from pancreatoduodenectomy until removal of last abdominal drain, median (range), days	44 (17–188)
Comprehensive complication index, median (range)	54.0 (39.7–89.1)
Post pancreatectomy hemorrhage, *n* (%)	5 (29)
Delayed gastric emptying, *n* (%)	5 (29)
Pulmonary embolism, *n* (%)	2 (12)
90-day mortality, *n* (%)	0 (0)
In-hospital mortality, *n* (%)	1 (6)
Length of stay, median (range), days	41 (34–135)
Follow-up time, median (interquartile range, months)	27.0 (12.2–93.7)
Postoperative new-onset exocrine insufficiency, *n* (%)	16 (94)
Postoperative new-onset endocrine insufficiency, *n* (%)	5 (29)
Insulin dependent diabetes mellitus	4
Oral medication (Metformin) diabetes mellitus	1

Percentages might not sum to 100 as a result of rounding.

^a^ Circulatory failure.

^b^ Respiratory and circulatory failure in two patients. Respiratory and kidney failure in one patient.

^c^ CT findings in 6 of the 7 patients: fluid collection around PJ (*n* = 2), fluid and air collection around PJ (*n* = 1), disrupted PJ (*n* = 1), necrosis in PJ (*n* = 1), ascites (*n* = 1).

^d^ All patients had abdominal drains at time of reoperation, either drains placed during the index operation or drains placed between index operation and reoperation. CRP, C-reactive protein; CT, computed tomography; PJ, pancreaticojejunostomy.

Overall the reoperation rate after pancreatoduodenectomy during the study period was 13.2% (*n* = 109). PDO was performed in 17 (15.6%) of the reoperations, whereas completion pancreatectomy was performed in 22 (20.2%). For numerical comparison, CCI was median 84.3 and 90-day mortality 22.7% in the 22 patients undergoing completion pancreatectomy. Main procedures performed in the remaining 70 reoperations were: control of intraabdominal bleeding (*n* = 32), redo or suture of hepaticojejunostomy (*n* = 10), drainage or suture of bile leak (*n* = 5), suture of wound dehiscence (*n* = 4), delayed reconstruction of the three anastomoses (*n* = 4), revascularization of portal vein or hepatic artery due to thrombosis (*n* = 4), small bowel resection or adhesiolysis due to small bowel obstruction (*n* = 3), redo gastrojejunostomy due to duodeneojejunostomy leak (*n* = 2), redo gastrojejunostomy due to obstruction (*n* = 2), suture of stomach perforation (*n* = 1), splenectomy due to spleen necrosis (*n* = 1), suture of pancreaticojejunostomy (*n* = 1), and colectomy due to bowel necrosis (*n* = 1). Twenty-one (30%) of these 70 patients had a POPF needing optimal drainage during the reoperation.

For historical comparison, between October 2015 and December 2023, 896 patients underwent pancreatoduodenectomy. Reoperation rate was 11.5% (*n* = 103), and of these POPF was the main cause in 30 patients. Completion pancreatectomy was performed in nine (1%) patients, and no patients were treated with PDO.

## Discussion

The current study demonstrates that PDO is a safe and feasible approach for managing severe POPF after pancreatoduodenectomy. However, 76% of patients still had a persistent POPF after undergoing PDO. Moreover, 29% of patients developed new-onset diabetes mellitus, and two patients developed operative complications.

Only a few studies have examined the use of PDO for the treatment of POPF after pancreatoduodenectomy (9). Balzano's findings did not confirm the potential benefits of PDO, as they found that PDO and simple drainage through relaparotomy led to similar outcomes (9). The high rate of persistent POPF (76%) after PDO in this study is of concern, as it necessitated prolonged drainage for a median of 35 days. During PDO, the dehiscent jejunal loop is resected to convert an “activated” POPF into a “pure” POPF, thus preventing the mixing of pancreatic enzymes with bilioenteric secretions. The debate surrounding whether to perform a completion pancreatectomy or a pancreas-preserving procedure during relaparotomy for POPF after pancreatoduodenectomy is ongoing, and the rates of completion pancreatectomy vary among different centers (5, 13). The rate of completion pancreatectomy in the current study of 2.7%, is similar to the rate reported in a large study conducted in Heidelberg of 3% (120 out of 3,953), but higher than the rate reported in a multicenter study conducted in the Netherlands of 0.74% (36 out of 4,877) (5, 13). Starting from October 2015 and until December 2023, completion pancreatectomy was performed in 1% of all pancreatoduodenectomies, PDO was discontinued, and there was a shift towards a more conservative strategies, in line with several other reports (1, 9).

The Dutch Pancreatic Cancer Group conducted a study in 2017 showing that percutaneous drainage as the initial intervention for severe POPF after pancreatoduodenectomy was associated with better clinical outcomes, including lower mortality, in comparison to primary relaparotomy (1). The authors attribute the success of percutaneous drainage to adherence to two main surgical principles: ensuring adequate source control and avoiding further harm. The in-hospital mortality rate was 14.1% (nine out of 64) in the Dutch study with a mini-invasive pancreas-preserving procedure, compared to 5.9% (one out of 17) in the current study with relaparotomy and PDO. The higher mortality rate may be attributed to a higher disease severity and a higher incidence of single or multi-organ failure in the Dutch study of 34.4% compared to 23.5% (four out of 17) in our study. It is important to acknowledge that comparison of such studies is challenging because the choice of treatment for severe POPF after pancreatoduodenectomy is both center and surgeon dependent (14). Still, it is noteworthy that the time interval from pancreatoduodenectomy until last abdominal drain could be safely removed was median 29 days in the Dutch study that utilized primary percutaneous drainage. In the current study, this timeframe was extended to 41 days (with a median of 35 days after PDO) which indicates that PDO does not completely heal the POPF.

Importantly, 29% of the patients developed postoperative new-onset diabetes mellitus after PDO. Mazzaferro showed that 36.7% of the patients developed new-onset diabetes after neoprene-based PDO during the primary operation in a selected group of pancreatoduodenectomy patients at high risk of POPF (7). Another study comparing total pancreatectomy with pancreatoduodenectomy in patients with high risk pancreatic anastomosis observed new-onset diabetes in 13% of the patients undergoing pancreatoduodenectomy (15). Thus, the rate of new-onset diabetes mellitus is seemingly higher in patients undergoing elective or emergent PDO compared to conventional anastomosis.

Some limitations of this study should be acknowledged. Firstly, this is a small, retrospective, single-center study with a high risk of selection bias. The indication for relaparotomy may differ depending on the surgeons' evaluation of the patients' condition, and the criteria for performing PDO or completion pancreatectomy may have differed among surgeons and from case to case. Secondly, no comparison was made with alternative approaches, as it was beyond the scope of this study to compare PDO with completion pancreatectomy or conservative strategies. There is a lack of consensus among surgeons on the indications for reoperations and the appropriate intraoperative approach during reoperations in cases of severe POPF following pancreatoduodenectomy (14). Further studies are needed to clarify the dilemma of whether or not to reoperate for a severe POPF following pancreatoduodenectomy, and if a relaparotomy is deemed necessary, which approach should be attempted. The use of PDO in the treatment of severe POPF after pancreatoduodenectomy has been discontinued in our institution, primarily due to pharmaceutical regulations making the glue unavailable for routine clinical purposes. A recent review found that the potential advantages of PDO remain questionable when compared to debridement and drainage (4). The findings of this study, which include persistent POPF and prolonged drainage, long-term exocrine and endocrine pancreatic insufficiency, and local complications after PDO, suggest that it is advisable to explore alternative pancreas-preserving techniques before considering PDO.

## Data Availability

The raw data supporting the conclusions of this article will be made available by the authors, without undue reservation.
